# Plasma uric acid and tumor volume are highly predictive of outcome in nasopharyngeal carcinoma patients receiving intensity modulated radiotherapy

**DOI:** 10.1186/1748-717X-8-121

**Published:** 2013-05-15

**Authors:** Hui Lin, Huan-Xin Lin, Nan Ge, Hong-Zhi Wang, Rui Sun, Wei-Han Hu

**Affiliations:** 1 Department of Radiation Oncology, Sun Yat-sen University Cancer Center, Guangdong Province, Guangzhou, 510060, China; 2 Surgical Intensive Care Unit, Xiangtan Central Hospital, Hunan Province, Xiangtan, 411100, China; 3 State Key Laboratory of Oncology in South China, Guangdong Province, Guangzhou, 510060, China

**Keywords:** Intensity-modulated radiotherapy, Nasopharyngeal carcinoma, Plasma uric acid, Tumor volume, Prognosis

## Abstract

**Background:**

The combined predictive value of plasma uric acid and primary tumor volume in nasopharyngeal carcinoma (NPC) patients receiving intensity modulated radiation therapy (IMRT) has not yet been determined.

**Methods:**

In this retrospective study, plasma uric acid level was measured after treatment in 130 histologically-proven NPC patients treated with IMRT. Tumor volume was calculated from treatment planning CT scans. Overall (OS), progression-free (PFS) and distant metastasis-free (DMFS) survival were compared using Kaplan-Meier analysis and the log rank test, and Cox multivariate and univariate regression models were created.

**Results:**

Patients with a small tumor volume (<27 mL) had a significantly better DMFS, PFS and OS than patients with a large tumor volume. Patients with a high post-treatment plasma uric acid level (>301 μmol/L) had a better DMFS, PFS and OS than patients with a low post-treatment plasma uric acid level. Patients with a small tumor volume and high post-treatment plasma uric acid level had a favorable prognosis compared to patients with a large tumor volume and low post-treatment plasma uric acid level (7-year overall OS, 100% vs. 48.7%, *P* <0.001 and PFS, 100% vs. 69.5%, *P* <0.001).

**Conclusions:**

Post-treatment plasma uric acid level and pre-treatment tumor volume have predictive value for outcome in NPC patients receiving IMRT. NPC patients with a large tumor volume and low post-treatment plasma uric acid level may benefit from additional aggressive treatment after IMRT.

## Introduction

Nasopharyngeal carcinoma (NPC) is a common epithelial malignancy in southern China [1,2]. NPC has a distinct epidemiology, etiology and clinical course compared to other head and neck squamous cell carcinomas. Intensity modulated radiotherapy (IMRT) has gradually replaced two-dimensional conventional radiotherapy (2D-CRT) as the primary means of radiotherapy, and has led to superior locoregional control and improved long-term survival rates in NPC patients. The 3-year overall survival (OS) rate for NPC patients treated by IMRT combined with cisplatin concurrent chemotherapy is currently 85-90% [3-6].

Uric acid is the end product of purine metabolism. Radiotherapy is associated with increased oxidative damage to DNA [7]. A high plasma uric acid level may occur due to increased purine metabolism by xanthine oxidase, as a consequence of RNA-DNA breakdown in patients receiving radiotherapy [7]^.^ The plasma uric acid level may be used as an indicator of radiosensitivity.

Recently, a number of reports have demonstrated the prognostic significance of the primary gross tumor volume for predicting local control, distant metastasis and overall survival in NPC patients receiving IMRT [8]; however, the prognostic value of plasma uric acid levels, or plasma uric acid levels combined with the primary tumor volume, remain unknown in NPC patients receiving IMRT .

In this study, the clinical records of NPC patients treated with radical IMRT at Sun Yat-sen University Cancer Center were retrospectively analyzed. The pre-treatment tumor volumes and post-treatment plasma uric acid levels were evaluated, with the aim of establishing prognostic subsets for the selection of appropriate treatment strategies in NPC.

## Materials and methods

### Clinical data

The study included 130 pathologically diagnosed NPC patients without distant metastases, who were treated with definitive-intent IMRT techniques at the Department of Radiation, Sun Yat-sen University Cancer Center between December, 2003 and December, 2005. The entry criteria consisted of: 1) a recorded post-treatment plasma uric acid level; 2) complete clinical data; 3) treated with IMRT; and 4) normal renal function.

The routine pre-treatment evaluation included a blood test, pre-treatment and post-treatment biochemical tests, full clinical examination, nasopharyngeal fiber mirror inspection, chest X-ray, ultrasound imaging of the abdominal region, nasopharyngeal MRI scan, general bone scan and Epstein-Barr virus (EBV) EA-IgA test. All patients with N3 disease also received a chest CT scan. This study was approved by the Research Ethics Committee of the Sun Yat-sen University Cancer Center.

We have check medical records of the patients in our study, and found that none of them had a hyperuricemia or Gout history. We found 45 patients had the record of Plasma uric acid level measured before treatment and none of them with hyperuricemia.

### Radiotherapy

Radiation was delivered with a linear accelerator (Varian, Palo Alto, CA, USA) using 6 MV photons. IMRT was delivered with a dynamic multileaf intensity-modulating collimator (PEACOCK-MIMiC system) using a slice-by-slice arc rotation approach. In accordance with our institutional treatment protocol, the primary tumor and upper neck above the cricoid cartilage (including levels II, III and VA) were treated with IMRT, whereas the lower neck (including levels IV and VB) and supraclavicular fossae were treated with a single anterior split field by conventional radiotherapy.

The CTV1 was defined as the nasopharyngeal gross tumor volume plus a 5–10-mm margin to encompass the high-risk sites of microscopic extension and the whole nasopharynx mucosa plus a 5-mm submucosal volume. The CTV2 was defined by adding a 5–10-mm margin to the CTV1 to encompass the low-risk sites of microscopic extension, the level of the lymph node located, and the neck area.

The prescribed doses were determined according to the simultaneous modulated accelerated radiation therapy boost technique [9]. The prescribed dose was 68 Grays (Gy) over 30 fractions to the nasopharyngeal gross target volume, 60 Gy/30 fractions to the define (CTV1), 54 Gy/30 fractions to the define (CTV2), and 60–66 Gy/30 fractions to the positive cervical lymph nodes. The prescribed dose to the lower neck and the supraclavicular fossae (irradiated with conventional radiotherapy) was 50 Gy/25 fractions for prophylactic intent and 60–66 Gy/30-33 fractions for therapeutic intent. Treatment plan was approved to meet the evaluation criterion of our institution previously reported by Xiao et al.^,^[3,8].

### Chemotherapy

In total, 93 of the 130 (71.5%) NPC patients received chemoradiotherapy; 86 of the 106 (81.1%) stage III-IVa patients received chemoradiotherapy. A total of 83 patients received concurrent chemotherapy with cisplatin (intravenous infusion of 30 mg/m^2^ every week, or intravenous infusion of 100 mg/m^2^ every 3 weeks) or paclitaxel (intravenous infusion of 30 mg/m^2^ every week). Reasons for deviation from the guidelines included age or organ dysfunction that suggested intolerance to chemotherapy.

### Pre-treatment tumor volume measurement

All target volumes (gross tumor volume, CTV1 and CTV2) and the critical adjacent organs were outlined slice-by-slice on the axial fused plain and contrast-enhanced CT images in the treatment planning system in combination with the MR images. The treatment plans were evaluated and approved by three or four radiation oncologists specializing in NPC. The treatment planning system was used to automatically reconstruct a three-dimensional image and calculate the volume of the tumor targets and critical organs prior to treatment. In this study, the tumor volume included the gross volume of the primary tumor and enlarged lymph nodes.

### Measurement of post-treatment plasma uric acid level

Plasma uric acid level was measured after RT using an Hitachi 7080 automatic biochemical analyzer.

### Observation and follow-up

Patients returned for follow-up appointments at least every three months during the first two years and every six months thereafter, until death. Every patient underwent an EBV EA-IgA test, routine hematological and biochemistry profiles, chest X-ray, ultrasound imaging of the abdominal region, and MRI or CT scan of the nasopharynx. Patients with suspected distant metastases underwent chest and abdominal CT scans, and general bone or PET-CT scans. Follow-up times were calculated from the end of radiotherapy until first event.

The date of last follow-up was July 1, 2012; median follow-up for the entire cohort was 87 months (range, 6–101 months). The following endpoints (time to first defining event) were assessed: overall survival (OS), progression-free survival (PFS) and distant metastasis-free survival (DMFS).

### Statistical analysis

OS, DMFS and PFS were calculated by the Kaplan-Meier method and analyzed using the log-rank test with SPSS 19.0 (SPSS, Chicago, IL, USA). The Cox regression model was constructed by forward selection (introduction = 0.05; removal = 0.1); *P* < 0.05 was considered statistically significant. Receiver operating characteristic (ROC) curve analysis was applied to determine the optimal tumor volume cutoff point.

## Results

### Patient characteristics and outcome

The median age of the NPC patients was 44 years (range, 18–76 years). Eighty eight of the 130 (67.7%) patients were men. Ninety-seven (91.9%) patients had good performance status (Karnofsky performance score ≧ 90). Thirty seven (28.5%) patients received radiotherapy alone, and Ninety three (71.5%) patients received combined chemoradiotherapy. The T and N stage distributions of the 130 patients according to the 7th edition of the UICC staging criteria are shown in Table 1.

**Table 1:** Baseline clinicopathological characteristics of the 130 nasopharyngeal cancer patients

**Variable**	**Number of patients**	**%**
Gender		
Male	88	67.7
Female	42	32.3
Age		
< 45 years	61	53.1
≥ 45 years	69	46.9
Karnofsky performance score		
< 90	11	8.5
≥ 90	119	91.5
T category*		
T1/T2/T3/T4	13/19/70/28	10/14.6/53.8/21.5
N category*		
N0/N1/N2/N3	31/40/53/6	23.8/30.8/40.8/4.6
Stage*		
I/II/III/IVa	7/17/73/33	5.4/13.1/56.2/56.2
Chemotherapy		
Radiotherapy alone	37	71.5
Chemoradiotherapy	93	28.5

* According to the 7th edition of the American Joint Committee on Cancer/International Union Against Cancer staging system.

OS data was collected for 130 patients up to July 1, 2012, with a median follow-up time of 87 months (range, 6–101 months) and follow-up rate of 100%. By the end of follow-up, 23/130 (17.7%) patients had developed recurrence or metastasis; 4 (3.1%) had developed local recurrence, 9 (6.9%) had developed regional recurrence, and 10 (7.7%) had developed distant metastases including three cases of pulmonary metastasis, three cases of bone metastasis, one case of liver metastasis, one case of brain metastasis, and one case of pleural and peritoneal metastasis. In total, 21/130 (11.0%) of the patients died, 20 (9.6%) of whom died due to tumor progression. For the entire cohort, the 7-year OS, PFS and DMFS rates were 81.7%, 87.5% and 91.9%, respectively.

### Relationship between tumor volume and patient outcome

**Figure 1:**
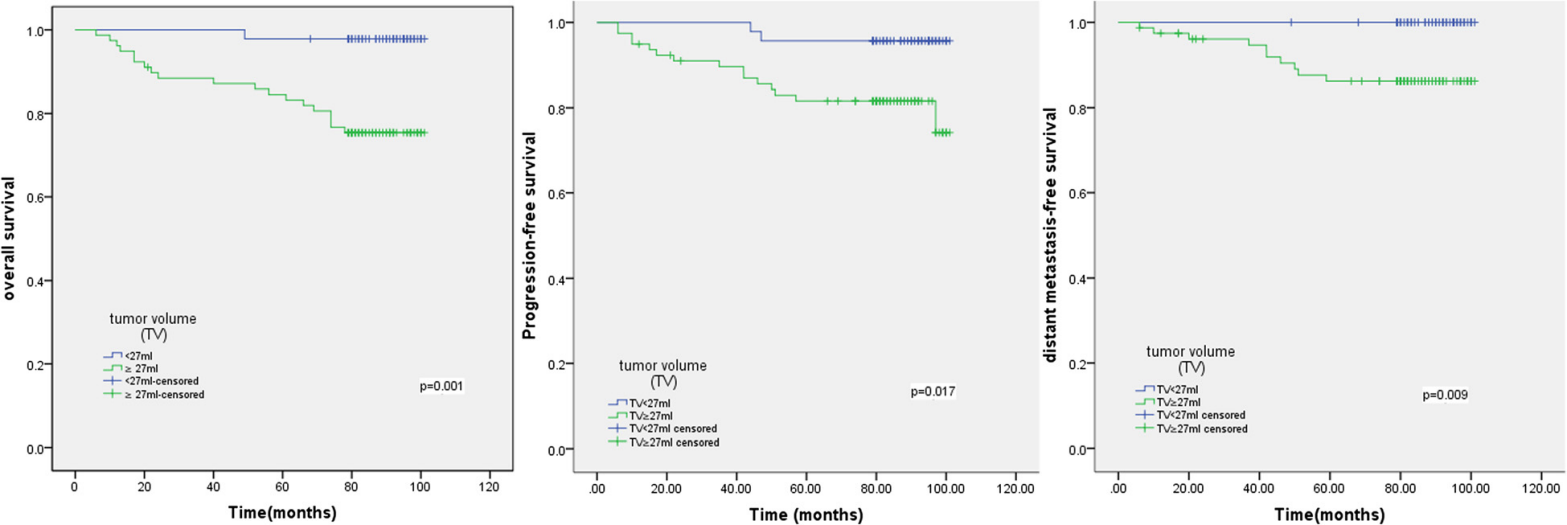
**Kaplan**-**Meier overall survival**, **progression**-**free survival and distant metastasis-free survival curves for 130 nasopharyngeal cancer patients treated with IMRT stratified by pre-treatment primary tumor volume.**

The median pre-treatment tumor volume for the 130 patients was 37.33 mL (range, 1.62–228.05 mL). Using ROC curve analysis, it was determined that 27 mL was an appropriate cutoff point for OS. The 7-year DMFS, PFS and OS rates for patients with a pre-treatment tumor volume smaller than 27 mL were significantly higher than patients with a pre-treatment tumor volume equal to or larger tumor volume than 27 mL (OS, 97.8% vs. 71.1%, *P* < 0.001; PFS, 95.7% vs. 81.6%, *P* =0.017; DMFS, 100% vs. 86.2%, *P* = 0.009; Figure 1).

### Relationship between plasma uric acid and patient outcome

**Figure 2:**
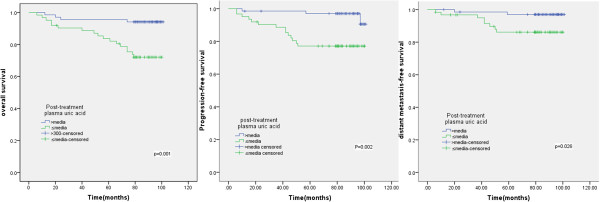
**Kaplan-Meier overall survival, progression-free survival and distant metastasis-free survival curves for 130 nasopharyngeal cancer patients treated with IMRT stratified by post-treatment plasma uric acid level.**

The median post-treatment plasma uric acid level for all patients was 301 μmol/L (range, 78–624 μmol/l). The 7-year DMFS, PFS and OS rates for patients with a post-treatment plasma uric acid level above the median value were significantly higher than patients with a post-treatment plasma uric acid level equal to or below 301 μmol/L (OS, 93.7% vs. 66.3%, *P* < 0.001; PFS, 90.5% vs. 77.2%, *P* = 0.002, DMFS, 97% vs. 86.1%, *P* = 0.026; Figure 2).

### Relationship of combined post-treatment plasma uric acid level and tumor volume with patient outcome

**Figure 3:**
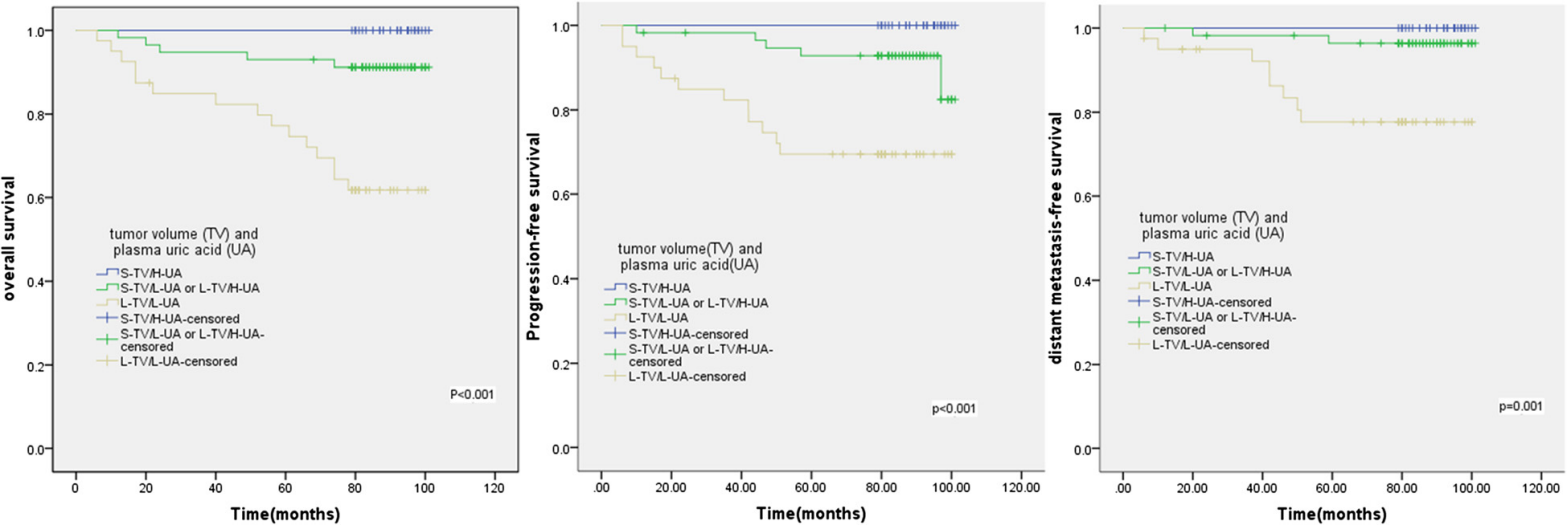
**Kaplan-Meier overall survival, progression-free survival and distant metastasis-free survival for 130 nasopharyngeal cancer patients treated with IMRT, stratified according a combination of pre-treatment primary tumor volume (TV) and post-treatment plasma uric acid (UA).** S-TV/H-UA indicates a small tumor volume (< 27 mL) and high post-treatment plasma uric acid level (>301 μmol/L); L-TV/L-UA indicates a large tumor volume (≥ 27 mL) and low post-treatment plasma uric acid level (≤301 μmol/L).

Patients were divided into three subgroups according to the post-treatment plasma uric acid levels and pre-treatment tumor volumes: (1) small tumor volume (< 27 mL) and high post-treatment plasma uric acid level (>301 μmol/L; *n* = 28 patients); (2) small pre-treatment tumor volume (< 27 mL) and low post-treatment plasma uric acid level, or large pre-treatment tumor volume (≥ 27 mL) and high post-treatment plasma uric acid level (>301 μmol/L; *n* = 60 patients); and (3) large pre-treatment tumor volume (≥ 27 mL) and low post-treatment plasma uric acid level (≤301 μmol/L; *n* = 35 patients). The 7-year OS rates for these three subgroups were 100%, 90.5% and 48.7%, respectively (p<0.001). The 7-year PFS rates in the three subgroups were 100%, 92.8% and 69.2%, respectively (p<0.001). The 7-year DMFS rates in the three subgroups were 100%, 96.4% and 77.7%, respectively (p=0.001) (Figure 3).

### Multivariate analysis

Multivariate analysis was performed to adjust for various prognostic factors. Post-treatment plasma uric acid level and pre-treatment tumor volume were the only independent predictors of OS. Both the post-treatment plasma uric acid level and tumor classification were identified as independent prognostic factors for PFS. Age, sex and node classification had no statistically significant effect on survival (Tables 2 and 3).

**Table 2:** Multivariate analysis of variables correlated with overall survival in 130 nasopharyngeal cancer patients treated with IMRT

**Variable**	**HR**	**95% CI**	***P *value**
Tumor volume, ≥ 27 mL vs. < 27 mL	12.543	1.315-119.641	0.028
Post-treatment uric acid, ≤301 μmol/L vs. > 301 μmol/L	6.858	2.065-22.775	0.002
N category*	0.908	0.478-1.725	0.768
T category*	1.102	0.506-2.400	0.806
Age, ≤ 45 years vs. > 45 years	0.645	0.254-1.635	0.355
Sex, male vs. female	0.376	0.113-1.251	0.111

HR, hazard ratio; CI, confidence interval; * According to the 7th edition of the American Joint Committee on Cancer/International Union Against Cancer staging system.

**Table 3:** **Multivariate analysis of variables correlated with progression**-**free survival in 130 nasopharyngeal cancer patients treated with IMRT**

**Variable**	**HR**	**95% CI**	***P *value**
Tumor volume, ≥ 27 mL vs. < 27 mL	1.540	0.259-9.167	0.635
Post-treatment uric acid, ≤301 μmol/L vs. > 301 μmol/L	4.852	1.334-17.650	0.017
N category*	1.157	0.570-2.348	0.687
T category*	3.048	1.152-8.068	0.025
Age, ≤ 45 years vs. > 45 years	0.337	0.112-1.014	0.053
Sex, male vs. female	0.748	0.245-2.287	0.611

HR, hazard ratio; CI, confidence interval; * According to the 7th edition of the American Joint Committee on Cancer/International Union Against Cancer staging system.

## Discussion

Various studies have attempted to identify predictive factors for poor patient outcome in NPC, by analyzing traditional clinical, pathological and molecular biomarkers as probable prognostic factors. Radiotherapy is associated with increased oxidative damage to DNA [10]. A high plasma uric acid level may occur in patients undergoing radiotherapy due to increased purine metabolism by xanthine oxidase, as a consequence of tumor cell RNA-DNA breakdown [11]. Patients with poorly differentiated tumors, such as Burkitt's lymphoma, may have an elevated uric acid level as a result of the rapid destruction of malignant cells. The plasma uric acid level can be used as an indicator of radiosensitivity. However, the predictive value of plasma uric acid levels has not previously been investigated in NPC patients treated with IMRT.

In the present study of a relatively large cohort of NPC patients treated with IMRT, we demonstrated that the post-treatment plasma uric acid level was a strong predictor of OS, PFS and OS. Patients with a post-treatment plasma uric acid level above 301 μmol/L had significantly better 7-year DMFS (93.7% vs. 66.3%, *P* <0.001), PFS (90.5% vs. 77.2%, *P* = 0.002) and OS rates (97% vs. 86.1% *P* = 0.026) than patients with post-treatment plasma uric acid levels equal to or below 301 μmol/L.

Ames et al. [12] reported that uric acid could protect both erythrocyte membranes and intact erythrocytes from lysis. Furthermore, uric acid may also protect longer-lived T and B lymphocytes and macrophages. It is reasonable to hypothesize that post-treatment plasma uric acid levels may exert a similar protective effect in NPC patients, which may provide a mechanism to explain the relationship between favorable outcome and high post-treatment plasma uric acid levels observed in this study. Further studies are needed to fully elucidate the mechanism by which a high post-treatment plasma uric acid level is linked to improved survival in NPC patients receiving IMRT.

Tumor volume is an important prognostic factor in cancer patients treated with primary radiotherapy [8]. It is well accepted that larger tumor volumes represent increased tumor clonogens and a larger hypoxic fraction, which may directly reduce the efficacy of radiation. Recently, a number of studies have demonstrated the prognostic significance of tumor volume for the evaluation of local control and OS in patients with NPC [13-16]. However, there have been few reports of the prognostic significance of tumor volume with respect to distant metastasis. Shen et al. [14] reported that tumor volume was not associated with the incidence of distant metastasis in NPC; however, distant failure-free survival was significantly related to N-classification, in accordance with Chua et al. [17]. Our study demonstrated that the 7-year DMFS, PFS and OS rates in patients with small pre-treatment tumor volumes (< 27 mL) were significantly higher than patients with pre-treatment tumor volumes equal to or larger than 27 mL. In this study, the tumor volume included the gross volume of the primary tumor and the enlarged lymph nodes; whereas Shen et al. [14] and Chua et al. [17] only included the gross volume of the primary tumor, and did not include the enlarged lymph nodes, which may explain the variation in the results of these studies. This study indicates that pre-treatment tumor volume is highly significant for the prediction of local control, distant metastasis and overall survival in NPC patients undergoing IMRT.

Furthermore, by combining the post-treatment plasma uric acid level and tumor volume, we identified three subgroups of patients. The subgroup of patients with a small pre-treatment tumor volume (< 27 mL) and high post-treatment plasma uric acid level (>301 μmol/L) had a favorable prognosis, with a 7-year overall survival rate of 100%. However, the subgroup of patients with a large pre-treatment tumor volume (≥ 27 mL) and low post-treatment plasma uric acid level (≤301 μmol/L) had a fairly poor prognosis, with a 7-year overall survival rate of 48.7%. This difference might be of clinical importance. The standard treatment for NPC is radiotherapy with or without concurrent chemotherapy, which can cure approximately 80% of the patients [18,19]. Our results were consistent with this, as the 7-year OS, PFS and DMFS rates for the entire cohort were 81.7%, 87.5% and 91.9%, respectively. The prognosis for NPC patients who develop metastasis is generally poor [20]. However, no specific indicator has yet been identified for this subgroup of patients. In the current study, we found that the prognosis of patients with a large pre-treatment tumor volume (≥ 27 mL) and low post-treatment plasma uric acid level (≤301 μmol/L) was poor, with a 7-year overall survival of 48.7%. This demonstrates that combination of the post-treatment plasma uric acid level and pre-treatment tumor volume has significant prognostic value in NPC, which may help to determine the optimal treatment approaches for high risk patients. Patients with a large tumor volume (≥ 27 mL) and low post-treatment plasma uric acid level (≤301 μmol/L) may benefit from newer, more aggressive treatment approaches, such as adjuvant chemotherapy and additional accelerated hyperfractionated radiotherapy.

Like many other retrospective studies in the literature, the interpretation of our results may be hampered by a number biases. Despite these limitations, the relatively large number of patients analyzed in this study suggests that the results are valid. In summary, the current study indicates that the post-treatment plasma uric acid level and pre-treatment tumor volume have predictive value for the outcome of NPC patients undergoing IMRT; however, these results need to be confirmed in larger, prospective clinical trials. This study indicates that NPC patients with a large pre-treatment tumor volume (> 27 mL) and low post-treatment plasma uric acid level (<301 μmol/L) should receive more aggressive treatment.

## Competing interests

The authors have declared no conflicts of interest.

## Authors’ contributions

Guarantors of integrity of the entire study: HL, W-HH; study concepts/study design: W-HH; data acquisition: NG, H-XL, W-HH; data analysis/interpretation: all authors; literature review: H-ZW, RS; statistical analyses: HL, NG, H-XL; manuscript drafting or revision for important intellectual content: HL, W-HH; manuscript final version approval: All authors read and approved the final manuscript.
